# Preliminary evaluation of the VIsion PERformance (VIPER) simulator

**DOI:** 10.1186/s40779-020-0231-8

**Published:** 2020-01-16

**Authors:** Denise S. Ryan, Rose K. Sia, Jennifer B. Eaddy, Lorie A. Logan, Jide O. Familoni, Hind Beydoun, Samantha B. Rodgers, Bruce A. Rivers

**Affiliations:** 1 Warfighter Refractive Eye Surgery Program and Research Center, Fort Belvoir Community Hospital, Fort Belvoir, VA 22060 USA; 2 Night Vision and Electronic Sensors Directorate, Fort Belvoir, VA 22060 USA; 3 Department of Research Programs, Fort Belvoir Community Hospital, Fort Belvoir, VA 22060 USA

**Keywords:** Functional visual performance, Military task performance

## Abstract

**Background:**

This study evaluated the VIsion PERformance (VIPER) simulator’s ability to assess the functional visual performance in warfighters conducting civilian and military tasks.

**Methods:**

Thirty service members, aged 25–35 years old with a best corrected distance visual acuity (VA) better than or equal to 20/20 or logarithm of the minimum angle of resolution (logMAR) 0.00, were randomized to locate and identify road signs and mock improvised explosive devices (IEDs) under either daytime conditions or with infrared imagery, with (cc) and without (sc) wearing their habitual correction. Participants also underwent binocular uncorrected (UDVA) and corrected (CDVA) visual assessment, refraction, contrast sensitivity testing and wavefront aberrometry.

**Results:**

The mean age was 28.47 years. The manifest spherical equivalent was − 3.16 ± 1.75 diopters (D), the UDVA in both eyes (OU) was logMAR 0.83 ± 0.47, and the CDVA OU was − 0.11 ± 0.06. For VIPER, the mean difference in the detection distance (DD) for road signs ccDD vs. scDD was 76.7 ± 52.8 m (*P* < 0.001). The average difference in identification distance (ID) ccID vs. scID was 13.9 ± 6.3 m (*P* < 0.001). The mean accuracies were 83.5 and 27.9% for cc and sc, respectively (*P* < 0.001). The regression analysis indicated that a 1.6 m change in the distance accounts for a 1% change in the accuracy (*P =* 0.002). Without correction, a 4.1 m change accounts for a 1% change in the accuracy (*P* < 0.001). The average IED ccDD was 29.9 ± 8.2 m, and that for scDD was 13.2 ± 13.6 m (*P* < 0.001). The average IED ccID was 32.2 ± 6.2 m and that for the scID was 7.4 ± 10.3 m (*P* < 0.001). The mean IED identification accuracy was 46.7 and 11.4% for cc and sc, respectively (*P* < 0.001).

**Conclusions:**

The preliminary results reflect VIPER’s ability to assess functional visual performance when detecting and identifying signs and IEDs. Furthermore, VIPER is able to detect performance changes with and without correction.

## Background

The visual function of an individual soldier on the modern battlefield has a direct and critical impact on the soldier’s decision-making process and has a potential impact on the unit and the mission outcome. Impairment of visual function resulting from visual aberrations is most evident under intermediate and low light conditions, which are often the prevailing condition in military operations [1–3]. Diminished viewing conditions exacerbate any existing visual dysfunction to create difficulties in object detection, discrimination, recognition and targeting.

The relationship between quality of vision and visual performance is complex and not perfectly understood. Quality of vision can be assessed by measuring the visual acuity (VA), the contrast sensitivity (CS), and the contrast sensitivity function (CSF). VA is the ability to resolve fine spatial details at a standard distance. The CS measures how well one distinguishes an object from the background, which is especially important in low light and reduced visibility conditions. Plotting the results of the CS at varying spatial frequencies (SFs) generates the CSF, an illustration of the performance threshold of the visual system [3–6].

Another subjective measure of the quality of vision is the dynamic VA, which is defined as an observer’s ability to resolve a target when either the target or observer is moving. These tests are based on an individual’s perception of what they see, rather than the image actually captured. Objective analysis of the quality of vision can be accomplished using wavefront aberrometry. Wavefront aberrometry measures the divergence of light as it passes through the eye. Vision errors can be expressed as a topographic map or as Zernike polynomials, each detailing an aberration of a specific wavefront point. Spherical aberrations, comas, and trefoils have been shown to be significant in quantitatively expressing aberrations affecting visual quality [7].

While the process of measuring visual function is continuously undergoing refinement, ongoing research determines how a person functions in vision-related activities. Visual function can be measured for each eye separately, whereas functional vision is not applied to the individual eye. It describes how a person’s integrated vision functions. Classic vision tests in an exam lane probe the threshold of identifying stimuli of various sizes and illumination and contrast levels. On the other hand, functional vision tests assess performance in complex environments in which multiple parameters may vary simultaneously in unpredictable combinations.

Researchers have developed a number of platforms for testing functional vision. Notable among them are driving simulators, and in a military context, performance on a shooting range [1, 8–11]. In a driving simulator, participants’ ability to detect and identify objects and road signs in scenery projected on a monitor are scored. At the shooting range, the score of a target can be used as a measure of functional vision [11]. Both functional performance tests are confounded by the required cognitive loading, which is the mental effort required to complete the associated task, and the individual’s ability [12]. Tasks requiring a heavier cognitive load are prone to increased error [12]. Functional vision tests may be confounded by competing cognitive loading sources, for instance adding the task of operating a vehicle during a driving simulation while conducting the task of identifying and recognizing objects or when testing complex tasks (such as target practice) [13]. The VIsion PERformance (VIPER) simulator was designed to limit the aforementioned issues of cognitive loading by eliminating the subject’s need to pilot a vehicle. The aim of this study is to explore VIPER’s ability to assess the functional visual performance in warfighters conducting civilian and military tasks in the hope of identifying a reliable simulation-based alternative test.

## Methods

The Institutional Review Board at Walter Reed National Military Medical Center granted approval prior to the initiation of this study (WRNMMC-2016-0005 [FBCH STUDY NUMBER 900012]). Written informed consent for clinical testing and VIPER were obtained after counseling on the risks and benefits of participation in the study. All research adhered to the tenets of the Declaration of Helsinki, and HIPAA compliance was maintained throughout the study.

Thirty active duty U.S. military personnel aged 25–35 years old with the best corrected distance VA of 20/20 (logMAR 0.00) or better in both eyes were randomized into the study. This range was selected to minimize the effect of the reported decline in the contrast sensitivity (CS) and VA as a function of age [4, 14, 15]. Such a decrease can degrade task performance [15]. The participants were required to detect and identify road signs and mock improvised explosive devices (IEDs) in separate tests, under either daytime conditions or with infrared imagery on the VIPER system, with and without correction. Participants also underwent VA, manifest refraction, low-contrast VA, wavefront aberrometry, and CSF testing to assess their clinical visual performance.

### VIPER simulator

**Fig. 1:**
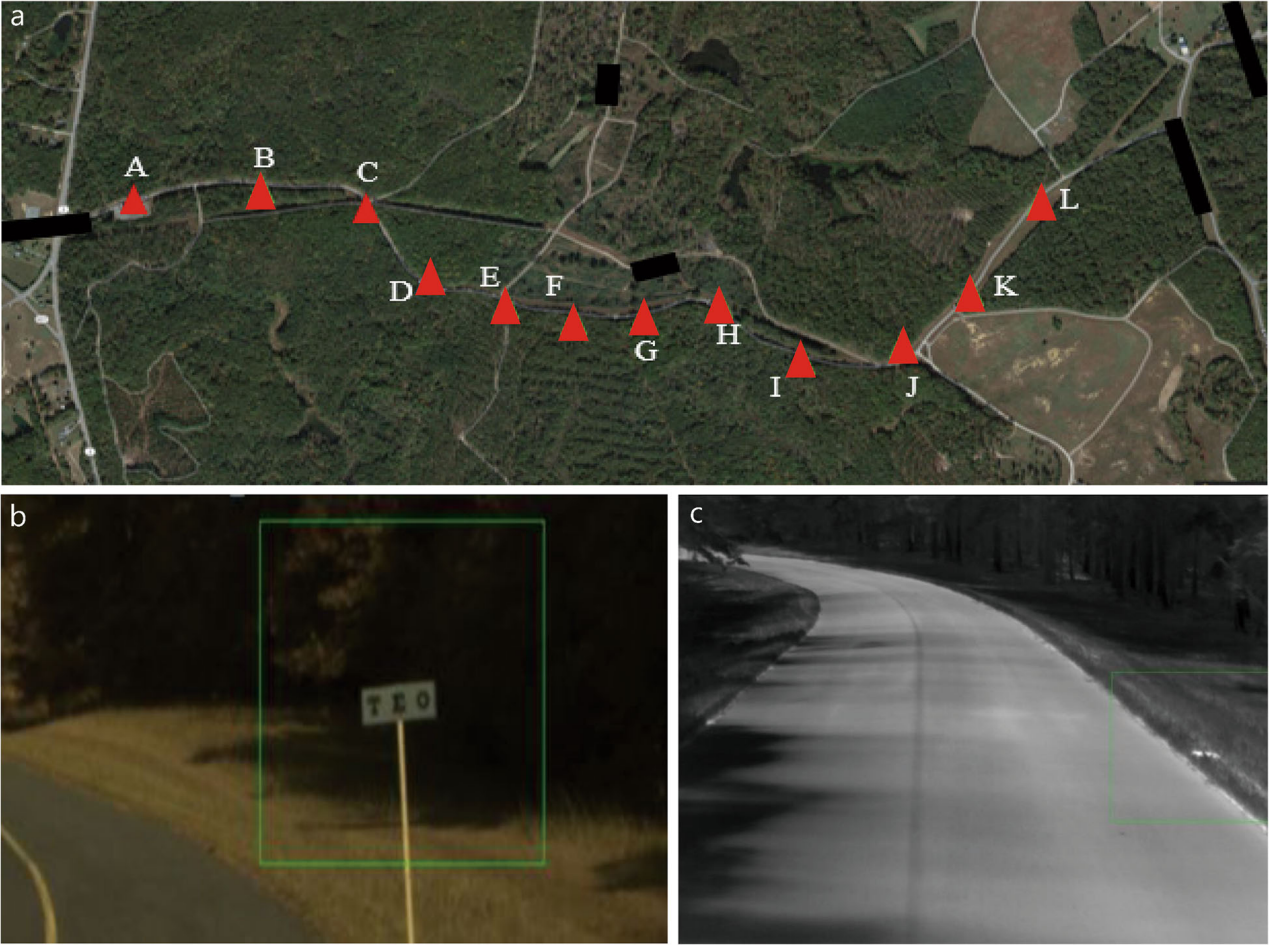
Examples of the VIPER course design (sign and improvised explosive device (IED) placement tagged with GPS coordinates) and examples of a simulation run in the visible (signs) and infrared (IEDs) spectra. **a** Illustration of the 1.1-mile data collection course for VIPER and location of signs and IEDs (6 each) along the course. **b** Example of a simulation run in the visible spectrum with the user indicating a sign detected inside the green box. (c) Simulation run in the infrared spectrum showing the image of a detected IED inside the green box

In developing VIPER, live-captured driving data were projected on a computer monitor to simulate the subject riding in a vehicle. The computer-based system tests and records the observer’s ability to detect and discriminate objects on the side of the road under varying vehicle speeds. To create the test course, a vehicle mounted with specialized cameras was driven along a long, flat, semi-curved road along which test objects were randomly placed. There were 2 sets of test objects: 6 signs, with a random selection of 3 letters, and 6 mock IEDs. The signs were designed to have the same relative spacing dimensions and character shapes as a Snellen chart, and their final size was determined by estimating the 70–80% identification range of 3 letters at approximately 80–100 m in the visible band. Dimensions were 8 × 24 in. for the signs, 4 × 4 in. for the characters, and with a spacing of 4 in. The paints used to make signs were chosen to have a good letter-to-background contrast in both the visible and long wave infrared (LWIR) spectra. Therefore, the background paint was a low-emissivity, aluminum-based paint, and the letter paint was a high-emissivity flat black paint. This combination gave the sign background a darker “cool” look in IR and brightness in the visible band, and the letters had a brighter “hot” look in the IR and were dark in the visible band. Six of the road signs were placed at randomly selected locations on the side of the roadway but were adjusted to permit visibility from afar. The IEDs were different kinds of deactivated land mines. The vehicle was driven at a GPS-monitored speed of 10 miles/hour during video capture. The signs were always placed along the right-hand side of the road, whereas the IEDs were placed at either edge of the road. GPS coordinates for the locations were logged (Fig. 1).

Data were captured at 10 mph along the course with 2 vehicle-mounted sensor systems: a 1080p HD color visible camera and an uncooled LWIR imager with a resolution of approximately 720, each running at 30 Hz with raw output. The sensors’ fields of view were chosen to match as closely as possible, and the relative aperture (F#) and integration time for the visible camera were chosen to maximize the dynamic range of the image for each run while allowing for the largest depth of focus, which usually meant that the F# was kept at F22, with adjustments made to the integration time as necessary. Each run was performed under the best lighting conditions possible for the course, with care taken to avoid backlit objects. However, there were cases where the objects were placed under a tree and subjected to shadowing.

Video files were read in, integrated onto the VIPER computer platform, and displayed with C^++^ code written in Microsoft Visual Studio. This program also collected the subject’s input for scoring. A graphical user interface (GUI) was written in the AutoIt scripting language to more easily configure the primary C^++^ program. The GUI permitted the viewer to choose operation in either visible or thermal wavebands; driving from east or west; at 10 or 30 miles per hour.

The participants undergoing VIPER testing were seated in front of a calibrated monitor at a distance of 36 in. with dimmed room illumination. The participants were shown a presentation to familiarize themselves with the task. Thirty participants were randomized into either the visual (luminance of 15.96 cd/m^2^) or infrared (luminance of 134.8 cd/m^2^) spectrum study. Each study included 4 different runs during which the distances at which the participant detected and identified road signs and IEDs with and without corrected vision were recorded. Each run took approximately 4 to 6 min for a total up to 24 min per participant for the 4 runs. If the subject was unable to see the monitor with uncorrected vision, the test was initiated and was allowed to run past 2 test objects before being canceled. The primary outcome measures were the average detection distance (DD) to a sign or IED and the average identification distance (ID).

VIPER also provided secondary outcome measures: the identification accuracy and the true detection rate. Each sign consisted of three Snellen characters that observers were required to identify in their correct order. The ID was logged if the observer correctly identified at least 1 of the 3 characters on the sign, with each correct letter assigned an accuracy of 33.3%. For IED identification, 5 choices were offered as a possible match. A correct match was logged as 100% accurate, and an incorrect match was logged as 0% accurate. The true detection rate was defined as the number of correctly identified road signs or IEDs divided by the total number of signs or IEDs (6). The false detection rate was defined as the number of incorrectly detected road signs or IEDs divided by the total number of detected road signs or IEDs (correctly and falsely detected). These secondary metrics measured the test subject’s sensitivity and selectivity.

### Clinical tests

High- and low-contrast visual acuity, CS, CSF, and wavefront aberrometry were assessed for each participant. Uncorrected (UDVA) and corrected distance visual acuities (CDVA) were measured using a 100% contrast Early Treatment Diabetic Retinopathy Study (ETDRS) chart (Precision Vision, Inc., LaSalle, Illinois) viewed at 4 m.

Low-contrast visual acuity testing was performed binocularly with best correction in an otherwise dark room using a 25% contrast Sloan chart (Precision Vision, Inc., LaSalle, Illinois) viewed at a distance of 4 m under mesopic conditions simulated by a chart viewed through a neutral density filter with screen luminance of 4 cd/m^2^. Correctly identified letters were given a value of − 0.02 logarithm of the minimum angle of resolution (logMAR); a line of five letters was equivalent to 0.1 logMAR.

High-contrast visual acuity and CS were also evaluated using the Rabin Super Vision Test (SVT) chart (Precision Vision, Inc., LaSalle, Illinois), with a screen luminance of 106 cd/m^2^. SVT visual acuity (SVT-VA) was assessed by altering the letter sizes (20/32 to 20/5 logMAR scale), while the SVT-CS was tested by adjusting the contrast of letters of a specific size (20/25; logMAR 0.10). When scoring the SVT-VA, a score of − 0.02 logMAR units was recorded for each letter correctly identified, whereas a score of 0.05 logarithm of the contrast sensitivity (logCS) units was calculated for each correct letter in the SVT-CS [16].

### Contrast sensitivity function (CSF)

The contrast threshold was assessed using the best corrected vision (trial frame) at 5 different SFs using Psykinematix (Cambridge Research Systems, Ltd., KyberVision, Japan), a display of calibrated visual stimuli of sinusoidal grating patterns with variable spatial frequencies viewed at 80 in. (2.03 m) and a mean luminance of 105 cd/m^2^. After a demonstration, the participant was required to choose the orientation of the stimuli on the screen. The contrast threshold was converted to CS and plotted for each SF to generate a contrast sensitivity function (CSF).

### Higher-order aberrations (HOAs)

Wavefront aberrometry was performed using the iDesign Advanced WaveScan Studio System (Johnson & Johnson, New Brunswick, New Jersey) and a Hartmann-Shack wavefront sensor. Under iDesign treatment mode, three optimal scans were captured for analysis. A wavefront diameter of 4 mm was used in the analysis. The total number of HOAs, comas, trefoils and spherical aberrations were reviewed.

### Participant confidence

Participants were asked to rate their confidence in using VIPER simulator on a scale of 0–10, with 0 indicating no confidence in performing the tasks and 10 indicating extreme confidence in performing the tasks [17].

Statistical analyses were performed using SPSS version 24.0 (IBM Corp, Armonk, New York). For continuous outcomes, paired *t*-tests were performed. Fisher’s exact tests were employed to compare outcomes for categorical variables. The eye with a better CDVA was selected for measures of the manifest spherical equivalent (MSE) and the total number of HOAs used in the regression models. Correlations between clinical and VIPER parameters were explored. Simple and multiple linear regression models were constructed for predictors of the DD for signs, the ID for signs, the DD for IEDs and the ID for IEDs. The following predictors were selected for entry into these models: CDVA, MSE, 25% mesopic contrast, SVT-VA, SVT-CS and total number of HOAs. Simple linear regression models were used to compute unadjusted slopes (beta coefficients), standard errors (SEs) and associated *P*-values for each separate predictor-outcome relationship. Using stepwise selection with thresholds for variable entry (*P* = 0.20) and variable removal (*P* = 0.21), multiple linear regression models were constructed to compute adjusted slopes (beta coefficients), SEs and associated *P*-values for predictors of each of the 4 outcome variables. Additionally, regression models were used to explore the associations of predictive factors of the ID, MSE, VA, sensor type, MSE, 25% mesopic contrast, SVT-VA, and SVT-CS on the accuracy. A *P*-value < 0.025 was considered statistically significant to adjust for two primary outcome variables.

## Results

**Fig. 2:**
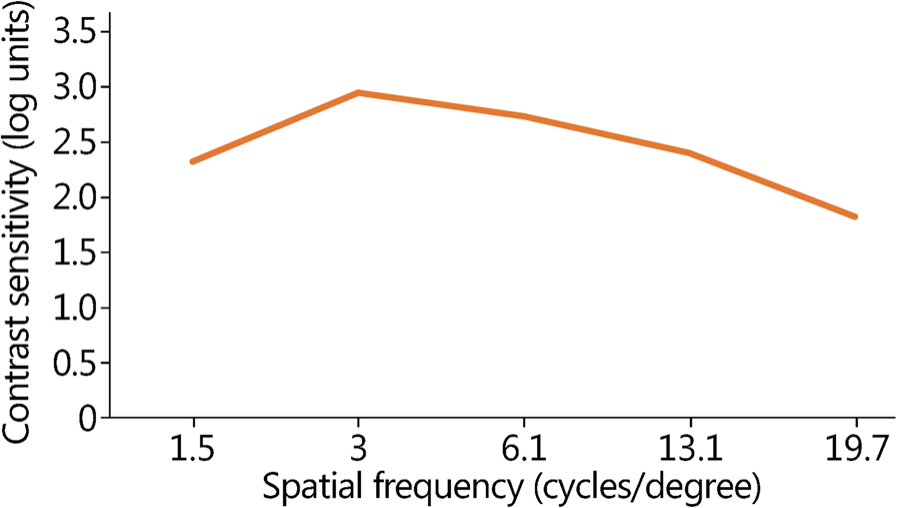
Mean contrast sensitivity over five spatial frequencies tested in this cohort

**Fig. 3:**
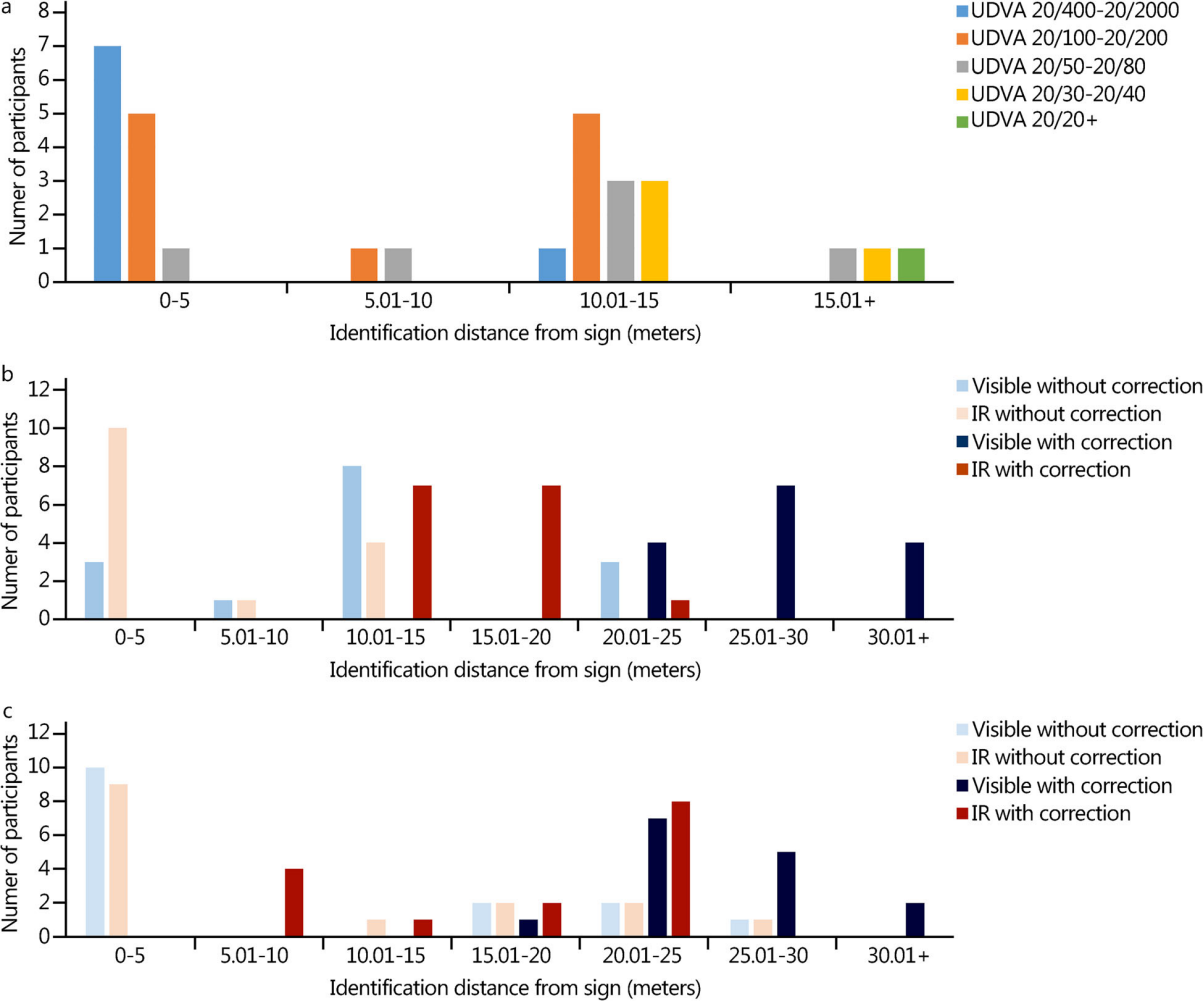
Identification distance relationship with corrected and uncorrected visual acuity and sensor type. **a** Presents the relationship between uncorrected distance visual acuity and the sign identification distance. **b** and **c** depict the relationship between the identification distance, sensor type and presence or absence of visual correction for signs (**b**) and IEDs (**c**)

Subject demographics and visual characteristics are presented in Table 1. The Super Vision Test VA and CS values were within normal limits [16]. The contrast sensitivity function was plotted, and peak sensitivity was observed at 3.0 cycles per degree in this cohort (Fig. 2). There was a significant correlation between the CS at 19.7 cycles per degree and Super Vision Test high-contrast visual acuity (*r* = − 0.432, *P* = 0.019). Participants’ functional visual performance on VIPER with and without correction is shown in Table 2. Figure 3a presents the relationship between uncorrected distance visual acuity and the sign identification distance. Figure 3b and c depict the relationship between the identification distance, sensor type and presence or absence of visual correction for signs (3b) and IEDs (3c).

**Table 1:** Overall subject demographics and characteristics (Mean ± SD)

Item	Value
Age (year)	28.5 ± 2.7
Uncorrected distance visual acuity, OU (logMAR)	0.83 ± 0.47
Corrected distance visual acuity, OU (logMAR)	−0.11 ± 0.06
Sphere, better eye (diopters, D)	−2.73 ± 1.69
Cylinder, better eye (D)	−0.86 ± 0.84
Manifest spherical equivalent, better eye (D)	−3.16 ± 1.75
Mesopic 25% low contrast acuity, OU (logMAR)	0.04 ± 0.08
Super Vision Visual Acuity, OU (logMAR)	−0.14 ± 0.06
Super Vision Contrast Sensitivity, OU (logCS)	1.17 ± 0.18
Higher order percent (%)	6.43 ± 4.62
Root Mean Square error (%)	2.03 ± 0.96
Spherical (μm)	0.01 ± 0.03
Coma (μm)	0.06 ± 0.03
Trefoil (μm)	0.05 ± 0.02

*OU* Both eyes, *D* Diopters, *logMAR* Logarithm of the minimum angle of resolution, *CS* Contrast sensitivity

**Table 2:** Comparison of VIPER functional performance with and without correction

	With correction	Without correction	*p*-value
Road signs			
Detection distance, m (mean ± SD)	117.6 ± 39.3	40.8 ± 44.3	<.001
*True detection rate, %	98.9 ± 4.2	73.3 ± 36.3	<.001
†False detection rate, % (mean ± SD)	34.7 ± 17.8	24.8 ± 26.6	.084
Identification distance, m (mean ± SD)	21.6 ± 6.7	7.6 ± 7.4	<.001
Accuracy, % (mean ± SD)	83.5 ± 18.1	27.9 ± 37.2	<.001
IEDs			
Detection distance, m (mean ± SD)	29.9 ± 8.21	13.19 ± 13.6	<.001
True detection rate, %	59.5 ± 17.8	22.2 ± 24.8	<.001
False detection rate, % (mean ± SD)	67.5 ± 19.9	46.1 ± 41.0	.008
Identification distance, m (mean ± SD)	32.2 ± 6.17	7.43 ± 10.25	<.001
Accuracy, % (mean ± SD)	46.67 ± 20.60	11.39 ± 17.30	<.001

$$ \ast \mathrm{True}\ \mathrm{detection}\ \mathrm{rate}=\frac{\mathrm{Correctly}\ \mathrm{detected}\ \mathrm{road}\ \mathrm{signs}\ \mathrm{or}\ \mathrm{IEDs}}{6\ \mathrm{sets}\ \mathrm{of}\ \mathrm{road}\ \mathrm{signs}\ \mathrm{or}\ \mathrm{IEDs}} $$

$$ \dagger \mathrm{False}\ \mathrm{detection}\ \mathrm{rate}=\frac{\mathrm{Falsely}\ \mathrm{detected}\ \mathrm{road}\ \mathrm{signs}\ \mathrm{or}\ \mathrm{IEDs}}{\left(\mathrm{Correctly}\ \mathrm{detected}+\mathrm{Falsely}\ \mathrm{detected}\ \mathrm{road}\ \mathrm{signs}\ \mathrm{or}\ \mathrm{IEDs}\right)} $$

The results of the simple and stepwise multiple linear regression models for CDVA, CDVA MSE, 25% mesopic contrast, Super Vision VA and CS, and total number of HOAs as predictors of the 4 outcomes (DD for signs, ID for signs, DD for IEDs and ID for IEDs) are shown in Tables 3, 4, 5 and 6. The beta coefficient is interpreted as the change in the outcome variable per unit change in the predictor variable. In the simple models, the unadjusted beta coefficient is estimated as the change in the outcome variable per unit change in the predictor variable. For the stepwise model, for the predictor variables selected, the adjusted beta coefficient is estimated as change in the outcome variable per unit change in the predictor variable, excluding nonpredictive variables from the model.

**Table 3:** Simple and multiple stepwise regression models for best corrected distance visual acuity logarithm of the minimum angle of resolution (logMAR), best corrected distance visual acuity manifest spherical equivalent (MSE), 25% mesopic contrast, super vision test LogMAR and logarithm of the contrast sensitivity (logCS), and total higher order aberrations (HOA) as predictors of average detection distance of signs (*n* = 29)

Item	Unadjusted				Adjusted^a^		
	*β*	SE	*P*	*R*^2^	*β*	SE	*P*
Best CDVA LogMAR	48.02	119.21	0.690	0.006	–	–	–
Best CDVA MSE	8.41	3.92	0.041	0.141	–	–	–
25% mesopic OU	45.62	88.14	0.609	0.009	–	–	–
Super vision visual acuity (LogMAR)	−4.22	121.60	0.973	< 0.0001	–	–	–
Super vision contrast sensitivity (log CS)	−18.59	40.13	0.647	0.008	–	–	–
Total HOA	3.17	1.48	0.042	0.144	3.17	1.48	0.042

^a^*R*^2^ = 0.144; “-”: Not selected for inclusion in stepwise regression model; *CDVA* Corrected distance visual acuity, *OU* Both eyes

**Table 4:** Simple (unadjusted) and multiple (adjusted) stepwise regression models for best corrected distance visual acuity logarithm of the minimum angle of resolution (logMAR), best corrected distance visual acuity manifest spherical equivalent (MSE), 25% mesopic contrast, super vision test LogMAR and logarithm of the contrast sensitivity (logCS), and total higher order aberrations (HOA) as predictors of average identification distance of signs (*n* = 29)

Item	Unadjusted				Adjusted^a^		
	*β*	SE	*P*	*R*^2^	*β*	SE	*P*
Best CDVA LogMAR	9.79	20.23	0.632	0.008	–	–	–
Best CDVA MSE	1.57	0.66	0.023	0.171	1.46	0.69	0.042
25% mesopic OU	−16.44	–	–	0.043	–	–	–
Super vision visual acuity (LogMAR)	−16.65	–	–	0.023	–	–	–
Super vision contrast sensitivity (log CS)	1.03	–	–	0.001	–	–	–
Total HOA	0.31	–	–	0.045	–	–	–

^a^*R*^2^ = 0.145; “-”: Not selected for inclusion in stepwise regression model; CDVA: Corrected distance visual acuity; OU: Both eyes

**Table 5:** Simple (unadjusted) and multiple (adjusted) stepwise regression models for best corrected distance visual acuity logarithm of the minimum angle of resolution (logMAR), best corrected distance visual acuity manifest spherical equivalent (MSE), 25% mesopic contrast, super vision test LogMAR and logarithm of the contrast sensitivity (logCS), and total higher order aberrations (HOA) as predictors of average detection distance of IEDs (*n* = 29)

Item	Unadjusted			Adjusted^a^			
	*β*	SE	*P*	*R*^2^	*β*	SE	*P*
Best CDVA LogMAR	3.73	25.00	0.882	0.001	–	–	–
Best CDVA MSE	1.91	0.81	0.025	0.166	1.58	0.84	0.070
25% mesopic OU	0.42	18.53	0.982	< 0.0001	–	–	–
Super vision visual acuity (LogMAR)	−34.24	24.60	0.175	0.065	−31.28	23.2623.2622.26	0.190
Super vision contrast sensitivity (log CS)	−3.94	8.40	0.642	0.008	–	–	–
Total HOA	0.26	0.33	0.450	0.022	–	–	–

^a^*R*^2^ = 0.191; Overall *P* = 0.064; “-”: Not selected for inclusion in stepwise regression model; CDVA: Corrected distance visual acuity; OU: Both eyes

**Table 6:** Simple (unadjusted) and multiple (adjusted) stepwise regression models for best corrected distance visual acuity logarithm of the minimum angle of resolution (logMAR), best corrected distance visual acuity manifest spherical equivalent (MSE), 25% mesopic contrast, super vision test LogMAR and logarithm of the contrast sensitivity (logCS), and total higher order aberrations (HOA) as predictors of average identification distance of IEDs (*n* = 29)

Item	Unadjusted			Adjusted^a^			
	*β*	SE	*P*	*R*^2^	*β*	SE	*P*
Best CDVA LogMAR	5.23	18.77	0.783	0.003	–	–	–
Best CDVA MSE	1.22	0.62	0.062	0.119	1.23	0.66	0.072
25% mesopic OU	−5.54	13.88	0.693	0.006	–	–	–
Super vision visual acuity (LogMAR)	−19.01	18.78	.320	0.035	–	–	–
Super vision contrast sensitivity (log CS)	−4.91	6.27	0.440	0.021	–	–	–
Total HOA	0.37	0.25	0.154	0.074	–	–	–

^a^*R*^2^ = 0.115; “-”: Not selected for inclusion in stepwise regression model; CDVA: Corrected distance visual acuity; OU: Both eyes

Road sign ID regression revealed that with correction, for every 1.6 m increase, there was a 1% change in the accuracy [*F*(1,29) = 15.03, *P* = 0.001]; without correction, for every 4.1 m increase, there was a 1% change in the accuracy [*F*(1,29) = 58.54, *P* < 0.001]. When the values of 0 for ID without correction were removed from the analysis, the ID relationship changed to a 6.7 m increase for a 1% change in accuracy, [*F*(1,16) = 15.09, *P* = 0.001]. When identifying IEDs, the regression analysis revealed that with correction, the ID relationship changed to a 1.9 m increase for a 1% change in accuracy [*F*(1,29) = 12.68, *P* = 0.001], whereas without correction, the ID relationship changed to a 1.5 m increase for a 1% change in accuracy [*F*(1,29) = 97.18, *P* < 0.001]. The regression was not significant when values of 0 for ID were removed (*P* = 0.48).

When considering the accuracy in identifying letters on signs with correction, stepwise regression found that the SVT-VA and sensor type significantly affected the performance (*P* = 0.001), with sensor type being strongly correlated (*P* < 0.001). A regression model for the accuracy in identifying letters on signs without correction was also constructed for the following predictors: UDVA, SVT-VA and CS, MSE, sensor type and average ID. The accuracy was significantly affected by the UDVA and average ID (*P* < 0.001), but only the average ID (*P* < 0.001) was significantly correlated. The accuracy of IED identification with correction was significantly impacted by the ID and MSE (*P* = 0.001), with the ID being significantly correlated (*P* = 0.008). Similarly, the accuracy of IED identification without correction was significantly affected with ID being a significant factor (*P* < 0.001).

The average participant confidence rating after testing on VIPER was 7.17 ± 2.35 (range: 3–10). There was a significant correlation between the confidence rating and accuracy of IED identification (*r* = 0.45, *P* = 0.014).

## Discussion

The VIPER simulator endeavors to assess functional visual performance as measured by the detection (DD) and identification distance (ID) to an object of interest. While visual acuity is an important test to establish ability to perform a job function, a review of occupational psychophysics recommends the use of simulations to recreate work environments as a means of gathering task performance data [17]. With increased usage of computer-generated imagery for readiness training and testing in the U.S. Army, immersive simulations evoking or replicating aspects of real-world experiences are bound to increase [18]. Detection and identification of objects or letters in a scene is intuitive as a method for measuring functional vision, hence the research on and development of simulated driving test platforms [1, 19]. Simulation-based training is also being used to measure competency and learning objectives. However, a review of occupational psychophysics suggests that a majority of studies do not measure how visual performance affects specific job tasks [17]. Additionally, assessing performance with both excellent and degraded vision may capture changes in performance related to changes in visual function [17].

The conditions under which the current study was conducted were photopic. Photopic conditions are only a portion of real-world scenarios in both civilian and military cases. Military engagements often occur at more extreme lighting and visibility conditions (night, dust, smoke, etc.). Similarly, the participants selected were between 25 and 35 years old, outside the range at which age-related contrast sensitivity (CS) and visual acuity (VA) decline should be significant. This specificity in the study design is intentional. In this proof-of-concept work, the authors sought to validate VIPER at photopic light levels and eliminate the decline in CS and VA as confounding factors. Measurements with the Super Vision Test found values within normal limits [16]. The Super Vision Test and 25% contrast test, sensitive to both over- and undercorrection, were performed to obtain the visual characteristics of the study cohort. In addition, the contrast sensitivity function supplied additional visual information on the participants undergoing testing. For example, glare can cause reduced sensitivity at a lower spatial frequency, while uncorrected vision may present as a reduction at a higher spatial frequency [20, 21]. Follow-up studies will extend these findings to more general lighting and extreme visibility conditions, a wider age range, and individuals who have undergone refractive surgery.

The preliminary results of VIPER show the ability to measure the DD and ID of signs and mock improvised explosive devices (IEDs). Additionally, VIPER was able to differentiate the performance with and without correction. As expected, the participants were able to identify letters significantly farther with correction than without correction, and the accuracy was significantly lower without correction when reading road signs (*P* < 0.001). Furthermore, participants were able to detect targets significantly farther with correction than without correction (*P* < 0.001).

The advantage of VIPER over other driving simulators is that it limits the cognitive load confounder associated with vehicle pilotage by requiring the subject to search, detect, and identify objects in autonomous moving scenery. However, the success of VIPER, similar to all search-and-detect algorithms, depends on the subject’s willingness to test the limits of their vision. Figure 3a illustrates this concept well; some participants in the same visual category, 20/50–20/80 for example, opted to identify signs in every identification range category. It is reasonable to expect that these participants, especially the subjects with lower correction needs, should have detected and identified signs at much farther distances. One can surmise that they are either nongamblers, who did not want to make a mistake in the detection or identification task, or they were not attentive when the signs became closer. Either case does not help detect the limits of their vision. To achieve the objective of effective functional vision testing and determine the threshold, initiating a forced choice methodology, similar to determining the contrast threshold used in this study, may show a performance benefit [22, 23]. When participants with a sign identification distance of 0 without correction were removed, the ID improved by 6.7 m, compared to the 4.1 m increase for each 1% improvement in accuracy. This effect was not observed when IED IDs of 0 were removed. A possible explanation may be due to the forced nature of the IED identification: five options were presented for identification, possibly encouraging participants to respond [23].

In a University of Leeds survey of driving simulators, the technical characteristics and applications of various simulators were reviewed [18]. The advantages of simulation research include cost and time savings and efficiency, experimental control and ease of data collection. The primary disadvantage was validity, both physical and predictive. Physical validity (biofeedback such as vibration and resistance) was a concern for the driving simulator, but not for the VIPER simulator. Rather, the concern with VIPER is predictive validity or how the participant performs. Figure 3b and c illustrate the range of ID accounting for both visual acuity and the sensor type. The different sensor types employed during daytime conditions miss the main advantage of thermal sensors – improved vision at nighttime or conditions in which there is a greater thermal differentiation. Planned future iterations of VIPER may increase the cognitive load by testing in extreme lighting conditions. To mitigate mental fatigue and impairment of visual function due to task length, VIPER testing will be designed to be time-limited [24, 25].

Another measure, the true detection rate, or the percentage of correctly detected signs/IEDs, was both significantly lower when subjects were tested without correction compared to with correction (*P* < 0.001), as expected. However, the false detection rate did not show a significant difference when the test was performed with or without correction when detecting road signs (*P* = 0.084). As previously mentioned, this finding may be attributed to the user not wanting to gamble. There was a significant difference in the false detection rate with or without correction for detecting IEDs (*P* = 0.008). This finding may be due to the task itself. The profiles of IEDs are low. Therefore, their search and detection are difficult, causing participants to be more cautious.

Another concern in the assessment of functional vision is the level of realism imparted by the task, as well as the ability to isolate and measure functional vision. Simulation-based testing can measure a participant’s performance skills while minimizing the risks. A U.S. Army-sponsored study comparing the efficacy of computer-generated versus real-world visual perception revealed an underestimation in the distance, height, and speed perceived in computer-generated reality [26]. A limitation in the study by Gainer and Hiller was the failure to replicate real-world perception due to image display and content deficiencies [26]. Advances in technology and virtual reality are closing the gap in content and image display deficiencies. Recognition of combatants, a self-paced training module developed by the U.S. Army incorporates thermal imagery in a cost-effective software package deployable on multiple user-driven media devices, ensuring repeatable training pertinent to the military. As part of the NVESD Modeling and Simulation Division, standardized training incorporates up-to-date lessons learned and real imagery for training in military performance tasks to include detection and discrimination [27]. Validation studies have shown that the performance of trained observers in a field environment can accurately be represented with models that have been developed using laboratory-based human performance [28–32].

As part of the assessment of visual performance in the evaluation of new medical products, Drum et al. recognized the difficulty of eliminating or controlling for nonvisual factors such as noise or sensory feedback when evaluating visual performance in driving [1]. It was noted that real-world visual function assessment is hampered by cost and limited standardization, thereby promoting clinical tests for visual acuity, low-contrast acuity, contrast sensitivity, glare testing, etc. [1]. The clinical tests in this study show that the study population was within a normal range when reviewing the corrected distance visual acuity, contrast sensitivity and aberrometry results [31, 32]. Additionally, the age range of the study population was selected to limit age-related contrast sensitivity decline [14, 15]. Higher-order aberrations (HOAs) measured in this cohort are consistent with measurements in normal myopic eyes, but it should be noted that HOAs can positively or negatively affect visual performance [33–35].

In the review of the National Advanced Driving Simulator, clinical vision tests were compared to the vision performance during simulated night driving, and the studies found a correlation between the clinical outcomes and object recognition [36]. More research is recommended to develop improved evaluation methods. The regression analysis (Table 3) showed that there were no significant associations with the average DD for signs in the unadjusted or fully adjusted model. By contrast, the manifest spherical equivalent (MSE) (*β* = 1.57, *P* = 0.023) was significantly and positively associated with the average ID for signs in the unadjusted model but not in the adjusted model (Table 4). Similarly, the MSE was significantly and positively associated with the average DD for IEDs in the unadjusted model (*β* = 1.91, *P* = 0.025) but not in the adjusted model, which included the MSE (*β* = 1.58, *P* = 0.070) and SVT-VA (*β* = − 31.28, *P* = 0.190, Table 5). Notably, visual outcomes were not significant in performance, but the level of correction was pertinent: higher levels of myopia were associated with a shorter DD and ID. This finding may be due to a minimization effect from correcting higher levels of myopia [37]. None of the hypothesized predictors of the average ID for IEDs were significantly associated with this outcome in the unadjusted and adjusted models (Table 6). The *R*^2^ for the multiple linear regression models ranged between 22 and 40%, suggesting that the selected predictors explained a small percentage of the variance in the outcomes under study.

In addition, in the regression models for the accuracy of identifying road signs and IEDs with and without correction, significant correlations found the ID affected the accuracy in three out of the four scenarios. However, in identifying road signs with correction, the sensor type was correlated with the accuracy. This finding may be due to the photopic condition of the task as discussed earlier and not being able to take advantage of the thermal sensor. Future iterations of VIPER will include conditions in which the thermal sensor can be employed. The regression models did not include the visual acuity and contrast as predictors. Similarly, a previous study of aircraft recognition and detection showed that the contrast sensitivity function is not associated with the performance [38].

Previous studies of military vision and task performance show that degraded conditions impede detection and identification, but testing is costly, and the results may be confounded by an individual’s ability or familiarity with the task [9, 10, 39, 40], which may be a limitation of the IED component of VIPER. While riding in a military vehicle may be commonplace, recognition of IEDs and identification training may be limited. A further limitation is the confidence rating. Rather than an overall confidence rating, confidence should have been rated after each run to differentiate between test runs. The results show that the confidence rating and IED identification were weakly correlated (*P* = 0.028). IED identification was the more difficult of the two tasks, suggesting that the overall confidence rating may be based on the performance on the more difficult task. As practice and repetition have been shown to improve performance, a longer pretest trial may be recommended for future studies [17]. However, this concept must be balanced with task timing and complexity, as studies have shown that visual function is sensitive to mental and physical fatigue [24, 25, 41].

## Conclusions

We currently live in a world that defies simulation. However, this study found that VIPER was able to assess functional performance in a simulation that recreated environmental conditions that may be experienced by service members as a means of gathering task performance data. Preliminary results reflect VIPER’s ability to assess functional performance when detecting and identifying signs and IEDs. Future studies will assess the results before and after corrective eye surgery to test the ability of VIPER to discriminate more nuanced vision changes after surgery.

## Data Availability

The datasets used and/or analyzed during the current study are not publicly available. Access from the corresponding author’s institution can be requested by completion and approval of a Data Sharing Agreement Application.

## Abbreviations

CDVA: Corrected distant visual acuity
CS: Contrast sensitivity
CSF: Contrast sensitivity function
DD: Detection distance
ETDRS: Early Treatment Diabetic Retinopathy Study
GUI: Graphical user interface
HOAs: Higher-order aberrations
ID: Identification distance
IEDs: Improvised explosive device
logCS: Logarithm of the contrast sensitivity
logMAR: Logarithm of the minimum angle of resolution
LWIR: Long wave infrared
MSE: Manifest spherical equivalent
SE: Standard error
SF: Spatial frequency
UDVA: Uncorrected distant visual acuity
VA: Visual acuity
