# Correction to: Preliminary evaluation of the Vision PERformance (VIPER) simulator

**DOI:** 10.1186/s40779-020-00276-2

**Published:** 2020-10-18

**Authors:** Denise S. Ryan, Rose K. Sia, Jennifer B. Eaddy, Lorie A. Logan, Jide O. Familoni, Hind Beydoun, Samantha B. Rodgers, Bruce A. Rivers

**Affiliations:** 1 Warfighter Refractive Eye Surgery Program and Research Center, Fort Belvoir Community Hospital, Fort Belvoir, VA 22060 USA; 2 Night Vision and Electronic Sensors Directorate, Fort Belvoir, VA 22060 USA; 3 Department of Research Programs, Fort Belvoir Community Hospital, Fort Belvoir, VA 22060 USA

**Correction to: Military Med Res 7, 2 (2020)**

**https://doi.org/10.1186/s40779-020-0231-8**

**Correction**

Following publication of the original article [1], the editor identified the Tables 5 and 6 are incorrect. The correct Tables 5 and 6 are as below.

**Table 5:** Simple (unadjusted) and multiple (adjusted) stepwise regression models for best corrected distance visual acuity logarithm of the minimum angle of resolution (logMAR), best corrected distance visual acuity manifest spherical equivalent (MSE), 25% mesopic contrast, super vision test LogMAR and logarithm of the contrast sensitivity (logCS), and total higher order aberrations (HOA) as predictors of average detection distance of IEDs (*n* = 29)

Item	Unadjusted				Adjusted^a^		
	*β*	SE	*P*	*R*^2^	*β*	SE	*P*
Best CDVA LogMAR	3.73	25.00	0.882	0.001	–	–	–
Best CDVA MSE	1.91	0.81	0.025	0.166	1.58	0.84	0.070
25% mesopic OU	0.42	18.53	0.982	< 0.0001	–	–	–
Super vision visual acuity (LogMAR)	− 34.24	24.60	0.175	0.065	−31.28	23.26	0.190
Super vision contrast sensitivity (log CS)	−3.94	8.40	0.642	0.008	–	–	–
Total HOA	0.26	0.33	0.450	0.022	–	–	–

^a^*R*^2^ = 0.191; Overall *P* = 0.064; “–”: Not selected for inclusion in stepwise regression model; *CDVA* Corrected distance visual acuity, *OU* Both eyes

**Table 6:** Simple (unadjusted) and multiple (adjusted) stepwise regression models for best corrected distance visual acuity logarithm of the minimum angle of resolution (logMAR), best corrected distance visual acuity manifest spherical equivalent (MSE), 25% mesopic contrast, super vision test LogMAR and logarithm of the contrast sensitivity (logCS), and total higher order aberrations (HOA) as predictors of average identification distance of IEDs (*n* = 29)

Item	Unadjusted				Adjusted^a^		
	*β*	SE	*P*	*R*^2^	*β*	SE	*P*
Best CDVA LogMAR	5.23	18.77	0.783	0.003	–	–	–
Best CDVA MSE	1.22	0.62	0.062	0.119	1.23	0.66	0.072
25% mesopic OU	−5.54	13.88	0.693	0.006	–	–	–
Super vision visual acuity (LogMAR)	−19.01	18.78	0.320	0.035	–	–	–
Super vision contrast sensitivity (log CS)	−4.91	6.27	0.440	0.021	–	–	–
Total HOA	0.37	0.25	0.154	0.074	–	–	–

^a^*R*^2^ = 0.115; “–”: Not selected for inclusion in stepwise regression model; *MSE* Manifest spherical equivalent, *CDVA* Corrected distance visual acuity, *OU* Both eyes
